# Associations of Blood Analysis with Feed Efficiency and Developmental Stage in Grass-Fed Beef Heifers

**DOI:** 10.3390/ani8080133

**Published:** 2018-08-02

**Authors:** Nara R. B. Cônsolo, Jasper C. Munro, Stéphanie L. Bourgon, Niel A. Karrow, Alan H. Fredeen, Janel E. Martell, Yuri R. Montanholi

**Affiliations:** 1 Departamento de Zootecnia, Universidade de São Paulo, Pirassununga 13635-900, Brazil; nara.consolo@hotmail.com; 2 AgSights, Elora, ON N0B 1S0, Canada; munrojasper@gmail.com; 3 Ontario Veterinary College, University of Guelph, Guelph, ON N1G 2W1, Canada; stephaniebourgon32@gmail.com; 4 Department of Animal Biosciences, University of Guelph, Guelph, ON N1G 2W1, Canada; nkarrow2@gmail.com; 5 Department of Animal Science and Aquaculture, Dalhousie University, Truro, NS B2N 5E3, Canada; alan.fredeen@dal.ca; 6 Animal Production, Welfare and Veterinary Sciences Department, Harper Adams University, Newport TF10 8NB, United Kingdom; janel.martell@gmail.com

**Keywords:** alkaline phosphatase, biomarker, hemoglobin, immunoglobulin, leukocyte, physiology indicator trait, potassium, pregnancy, residual feed intake, triiodothyronine

## Abstract

**Simple Summary:**

Individual cattle of identical developmental stage vary in their efficiency of feed utilization to achieve a similar productive performance in terms of growth rate and body composition upon accounting for breed, age and gestation stage. Technical issues to measure individual feed intake on the farm limits the identification of feed-efficient cattle. This creates a demand for indirect approaches to infer feed efficiency, such as blood parameters. Our study revealed differences in blood parameters when comparing grass-fed heifers classified as either efficient or inefficient. These differences were also influenced by the developmental stage of the heifers; some blood analytes had distinct relevance to infer about feed efficiency when comparing younger non-pregnant heifers with older and pregnant heifers. In general, improved feed efficiency seems to relate to a lower oxygen carrying capacity. We also provide evidence of associations between indicators of the immune system, blood enzymes and ions and feed efficiency. Additionally, blood analysis presented metabolic differences between non-pregnant heifers with older and pregnant heifers. Blood analysis as a practical measure for feed efficiency has relevance in the nutritional management and genetic improvement of beef cattle, which will contribute to the broad sustainability of beef farming.

**Abstract:**

Proxies for feed efficiency, such as blood-based indicators, applicable across heifers varying in genetic makeup and developmental state are needed. Assessments of blood analytes and performance were made in heifer calves and pregnant heifers. Residual feed intake, a measure of feed efficiency, was used to categorize each population of heifers as either efficient or inefficient. Efficient heifer calves had lower mean cell hemoglobin, greater lymphocyte count, and fewer segmented neutrophils at the end of the test compared to inefficient calves. Efficient pregnant heifers had greater counts of lymphocytes with fewer segmented neutrophils at the end than inefficient pregnant heifers. Efficient heifer calves exhibited higher specific immunoglobulin M than inefficient calves. Throughout the test, efficient heifer calves had elevated potassium and phosphorus, and reduced alkaline phosphatase (ALP) compared to inefficient heifers. Efficient pregnant heifers showed greater ALP, non-esterified fatty acids and creatinine, but lower cholesterol and globulin than inefficient pregnant heifers. Levels of red and white blood cells, creatine kinase, cholesterol, glucose, potassium and phosphorus were higher in heifer calves compared with pregnant heifers. There is potential for blood analytes as proxies for feed efficiency; however, it is necessary to consider the inherent associations with feed efficiency and heifers’ developmental stage.

## 1. Introduction

Feed is a major expense in beef cattle production, a large portion of which is attributed to the cow-calf herd and its high associated maintenance of retained energy [1]. The energetic trade-offs, inherent to beef farming, reflect in metabolic variability and open prospects to evaluate behavior, performance and physiological indicators, as reviewed by Rauw et al. [2]. As a result, individual animals will respond accordingly to their adaptation in a given production system, and in light of their experiences and developmental stage. Thus, understanding the physiological basis underlying the variation in feed utilization amongst animals may assist in identifying proxies for feed efficiency. This will enable further research to utilize the proxies for feed efficiency in the prediction and/or screening for earlier selection of replacement heifers, and to avoid costly and lengthy growth trials requiring the monitoring of feed intake. A practical measure to infer about feed efficiency is residual feed intake (RFI), which reflects the variation in the efficiency of feed utilization upon accounting for body size, weight and composition within a population [3]; therefore, reflecting the underlying variation in feed intake due to background metabolic requirements. Basically, a negative RFI indicates superior feed efficiency, as the animals was consuming less feed than the predicted and a positive RFI has inferior feed efficiency. Studies have identified potential proxies for RFI using traits associated with energy metabolism, including body heat dissipation [3], hepatic mitochondrial function [4] and visceral organ metabolism [5]. Research has also identified blood analytes as potential proxies for feed efficiency [6,7,8,9,10]. However, there is a need to further evaluate this broad class of assessments in replacement heifers across developmental stages, where animal selection for efficiency would provide timely information to cattlemen.

Blood cell parameters measured using the complete blood cell (CBC) analysis provide information about health status and metabolic state. Parameters such as red blood cell indices could be related to physiological functions, including oxygen consumption and transport, and may differ because of changes in metabolic rate [11]. Furthermore, white blood cell subpopulations can be associated with differences in immune function, which may also influence energy partitioning [12]. Positive relationships between feed efficiency and blood cell measures, including mean corpuscular volume, mean cell hemoglobin, and lymphocyte count have been observed in steers [10]. These associations have also been shown in other species, with higher concentrations of red and white blood cells and reduced mean corpuscular volume in feed-inefficient ewes [13].

The maintenance of the immune system is metabolically demanding, impacting nutrient and energy partitioning from growth, reproduction, and basal metabolism [14]. For instance, response to an infection can reduce protein accretion in muscle as amino acids are mobilized for glucose production, resulting in decreased productive performance [15]. Immunoglobulin M (IgM) and immunoglobulin G1 (IgG1) are primary responders of the humoral immune system in ruminants [16]. The responsiveness of the immune system can be objectively evaluated in cattle by measuring the specific immunoglobulin response to unfamiliar proteins, such as ovalbumin (OVA) [17]. This approach is effective for the study of health traits [18] and may serve to relate immune response and feed efficiency in the bovine.

Levels of blood plasma metabolites, including metabolic and inorganic ions, compounds, enzymes and hormones, are associated with energetically demanding functions that influence the efficiency of feed utilization. Metabolic products such as cholesterol [19], creatinine and non-esterified fatty acids (NEFA) [9] are thought to be related to feed efficiency in livestock. Similarly, ions related to cellular potential and acid/base status [20], and energy metabolism, including phosphorus, are associated with differences in feed efficiency [21]. Additionally, lower concentrations of triiodothyronine (T3) have been related to greater feed efficiency in beef cows [22], whereas in beef bulls, Bourgon et al. [6] observed an interaction between age and feed efficiency classes in the relationship between T3 and the efficiency of feed utilization.

Developmental stages, including growth and gestation influence hematological measures of cattle. Blood cell measures that appear to differ based on developmental stage include mean cell hemoglobin and mean corpuscular volume in heifer calves [10], and white blood cell count in pregnant heifers [23]. Immunoglobulins also vary according to the physiological stages of beef females, including IgM during weaning [24] and IgG during late pregnancy [25]. Metabolite blood profiles of heifers also differ in relation to age [7,26] and pregnancy status [7,27]. These differences have implications for the early-life prediction and robustness of potential proxies, affecting their practicality and relevance to the beef industry.

The simplicity of harvesting blood in cattle, the vast possibilities and relevance of this matrix to infer on complex traits, including those related to feed efficiency (here measured through RFI) and developmental stage, support further studies based on theoretical and/or exploratory analysis. We hypothesize that the evaluation of blood parameters in heifers of distinct developmental stages will broaden the understanding of the biology underlying feed efficiency. This may lead to the identification of blood constituents that could serve as proxies for feed efficiency. We also anticipate that developmental stage will play a role in the relevance of potential proxies for feed efficiency, justifying the comparison of heifers of distinct developmental stages. Therefore, the objectives of this study were to evaluate CBC parameters, specific IgG1 and IgM immune responses, and blood plasma metabolic profiles relative to feed efficiency (RFI) classification in forage-fed beef heifer calves and pregnant beef heifers, as well as the potential metabolic differences between the developmental stages of the heifers.

## 2. Materials and Methods

### 2.1. Animals and Experimental Design

Experimental procedures involving animals were performed in accordance with the recommendations of the Canadian Council on Animal Care guidelines [28]. The research protocol was approved by the Dalhousie University Animal Care Committee on 9 April 2014. Two groups of crossbred heifers consisted of 107 heifer calves (mean ± standard deviation), 287 ± 27.6 days of age, 253 ± 37.8 kg at the start of the trial), and 36 bred heifers (557 ± 93.5 days of age, 406 ± 41.7 kg) were consigned from 20 producers from the Atlantic region of Canada and housed at the Maritime Beef Test Station (Nappan, NS, Canada). Due to the inherent differences in farming and husbandry practices under which the heifers were raised, the following prophylactic treatments were given to all heifers prior to the performance evaluation: ivermectin (0.01 mL/kg Bimectin^®^ Bimedia, Oakbrook Terrace, IL, USA), vitamin E and selenium supplement (0.01 mL/kg Dystocel^®^, Zoetis, Kirkland, QC, Canada), tulathromycin (2.5 mg/kg Draxxin^®^, Zoetis, Kirkland, QC, Canada), bovine rhinotracheitis-virus diarrhea-parainfluenza-3-respiratory Syncytial virus vaccine (2 mL Bovi-Shield GOLD FP 5^®^, Zoetis, Kirkland, QC, Canada) and clostridium chauvoei-septicum-haemolyticum-novyi-sordellii-perfringens types C and D-haemphilus somnus bacterin-toxoid (2 mL Vision 8 Somnus with SPUR^®^, Merck Animal Health, Summit, NE, USA).

The 107 heifer calves were divided into two pens of 36 and one pen of 35, and 36 bred heifers were housed in a single pen. The heifers were maintained at a stocking rate of 15.1 ± 0.3 m^2^ per animal, and pens were bedded with wheat straw and included access to indoor feeding, as well as access to an outdoor yard. Heifers had a 14-day adaptation period to adjust to the automated feeding system, facilities and pen mates. Throughout the adaptation period and performance test, clinical signs and individual feed intake was monitored across all heifer to access their health status.

Heifers were tested for feed intake and productive performance for 124 days, from mid-June to the end of October (2014). Individual feed intakes were recorded using an automated feeding system (GrowSafe^®^ Feed Intake System, Airdrie, AB, Canada), with five feed bunks available in each pen. The automated feeding system recognized each heifer at the bunk by reading a radio-frequency identification ear tag and recording individual feeding events continuously through weight cells located in the base of the bunks. Heifers were weighed and scanned by ultrasound for body composition every 30.6 ± 3.0 days on four consecutive days (one pen per day). The pregnancy status of the 36 bred heifers was assessed by blood test pregnancy-specific protein B (BioPRYN^®^; Biotracking, Moscow, ID, USA) on day 28 of the performance evaluation and 31 were found to be pregnant. The five open heifers were kept with the pregnant heifers to maintain similar animal to bunk ratio and stocking rate. Heifers were also evaluated for immune response to OVA during days 56 to 77 of the productive performance evaluation.

Heifers were fed ad libitum a grass silage diet predominantly comprised of timothy, meadow fescue, bluegrass, reed canary grass, red clover and white clover. The total mixed ration was composed of 99.5% haylage, and 0.50% mineral and vitamin premix (containing 7.8% Na, 27% Ca, 0.02% P, 2.5% Mg, 2400 mg/kg Fe, 900 mg/kg Cu, 75 mg/kg iodine, 2300 mg/kg Mn, 2400 mg/kg Zn, 13 mg/kg Co, 3000 mg/kg Fl, 200,000 IU/kg vitamin A, 27,000 IU/kg vitamin D-3, 4000 IU/kg vitamin E.) on a dry matter (DM; %) basis. Feed samples were collected weekly and then pooled for analysis. The diet contained 39.4% of DM chemical composition of the diet (DM) basis was: crude protein 15.5%, acid detergent fiber 29.6%, neutral detergent fiber 53.7%, starch 6.5%, total digestible nutrients 70.3% and digestible energy 2.92 Mcal/kg.

Breed composition was determined via hair follicle DNA extraction and 50 K single-nucleotide polymorphism (SNP) sequencing (ADMIXTURE^®^ software, University of California, Los Angeles, CA, USA), followed by estimation of individual and population breed allele frequencies from the SNP data, using pairwise comparison [29]. The average breed composition of heifer calves was 28.5% Angus, 18.5% Simmental, 17.8% Limousin, 11.9% Hereford, and 23.3% other European breeds; and for pregnant heifers was 30.8% Simmental, 26.1% Hereford, 16.0% Angus, 8.6% Shorthorn, and 18.5% other European breeds.

### 2.2. Productive Performance and Biometrics

Body weights were measured between 0830 h and 1130 h prior to feeding, using a livestock scale (CattleMaster^®^, E. S. Martin Welding, Linwood, BC, Canada). Heifers were restrained in a squeeze chute (Pearson Livestock Equipment, Thedford, NE, USA) for ultrasound scanning. Ultrasound imaging was performed using an Aloka SSD-500^®^ ultrasound unit (probe model 5044; 172 mm; 3.5 MHz; Corometrics Medical Systems, Wallingford, CT, USA) as described by Montanholi et al. [3] to determine back fat thickness, rib eye area, marbling and rump fat thickness. Average ultrasound traits (back fat thickness (BKT; mm), rib eye area (REA; cm^2^), marbling (MAB; score 1: devoid; 11: prime) and rump fat thickness (RMP; mm)), body weight (BW; kg) and daily weight gain (ADG; kg/day) over the performance evaluation were determined using linear regression. The average individual DM feed intake (DMI; kg/day) over the performance evaluation test was computed by averaging the daily DM intake of each heifer. Feed to gain ratio (FG) was calculated by dividing the DMI by ADG. The age of the heifers at the end of the performance evaluation (AGE; days) was determined using records from their home farm herd data-keeping book. Days in gestation (DIG; days) of pregnant heifers was determined using the calf birth date and assuming a typical gestation length of 273 days.

The RFI (kg DMI/day) determination models were developed similarly to those described by Montanholi et al. [3] and Gonano et al. [7]. A liner regression was fitted to explain the variation on DMI based on the productive performance and biometric data outlined above for each population of heifers. The error term on these equations below represents the RFI (or the deviation between DMI observed and predicted). The model with the highest R^2^, while showing the lowest Bayesian information criteria, was selected for each population of heifers. The RFI of the heifer calves was calculated using the following model (R^2^ = 0.53):

DMI=−10.291+8.071(ADG)+0.040(BW)−0.103(REA)+0.118(RMP)+0.059(AGE)+error

Similarly, the RFI for pregnant heifers was calculated using the following model (R^2^ = 0.49):

DMI=−2.88+2.66(ADG)+0.017(BW)−0.018(REA)+0.081(BKT)−0.150(RMP)−0.11(MAB)+0.007(AGE)+0.005(DIG)+error

These regressions enabled us to calculate the predicted DMI for each heifer within each population. This determination served as basis to calculate RFI, which was given by the difference of observed DMI minus the predicted DMI on an individual heifer basis. 

### 2.3. Blood Sampling

Blood sampling was conducted between 0830 h and 1130 h prior to feed distribution. Heifers were restrained in the chute and secured with a nylon rope halter in order to expose the jugular vein region. The jugular area was disinfected with 70% isopropyl alcohol and blood samples were collected using 0.9 × 25 mm blood collection needles (BD Vacutainer^®^ Precision Glide, BD Inc., Franklin Lakes, NJ, USA). Blood was collected into sodium heparin tubes (BD Vacutainer^®^, BD Inc.) for the metabolite profile, EDTA tubes (Monoject^®^ Blood Collection Tube, Kendall Healthcare, Mansfield, TX, USA) for the CBC analysis, and serum separator tubes (BD Vacutainer^®^, BD Inc.) for the specific antibody response. Samples for CBC analysis were taken at the start and end of the performance evaluation, and stored at 4 °C prior to analysis. Samples for blood plasma metabolite profile analysis were taken every 30.6 ± 3.0 days during the performance evaluation, then stored at −80 °C until further processing. Blood samples for immune response were collected and kept at room temperature for 25 min to allow clotting before being further processed. Both the metabolite profile and the immune response samples were centrifuged at 4 °C at 3000× *g* for 25 min, then the supernatant was decanted into micro tubes and kept frozen until analysis.

### 2.4. Complete Blood Cell Profile

Blood cell parameters were measured with a hematology analyzer (Sysmex XT-20001 V Hematology Analyzer^®^, Sysmex Canada Inc., Mississauga, ON, Canada). Red blood cell parameters included red blood cell count (RBC; 10^6^ cells/µL), hemoglobin (g/dL), mean corpuscular volume (MCV; Hfl), mean cell hemoglobin (MCH; pg) and platelets (10^3^ cells/µL). White blood cell (WBC) parameters consisted of total white blood cell automated count (WBC; 10^3^ cells/µL) and, manual count of segmented neutrophils (% WBC) and lymphocytes (% WBC).

### 2.5. Blood Plasma Metabolic Profile

Concentrations of blood plasma metabolic enzymes including alkaline phosphatase (ALP; U/L), gamma-glutamyl transferase (GGT; U/L), aspartate aminotransferase (AST; U/L), creatine kinase (CK; U/L), glutamate dehydrogenase (GLDH; U/L); compounds including albumin (g/L), cholesterol (mmol/L), creatinine (CT; µmol/L), globulin (g/L), glucose (mmol/L), haptoglobin (g/L), non-esterified fatty acid (NEFA; mmol/L), urea (mmol/L); and ions including calcium (mmol/L), phosphorus (mmol/L), magnesium (mmol/L), sodium (mmol/L), potassium (mmol/L), chloride (mmol/L) and anion gap (mmol/L) were determined using an automated analyzer (Cobas^®^ c 311/501 analyzer, Roche Diagnostics GmbH, Indianapolis, IN, USA). Carbon dioxide levels were measured with an automated analyzer ((Hitachi) Cobas 4000 (c311^®^, Roche Diagnostics GmbH, Mannheim, Germany). Determination of *β*-hydroxybutyrate was carried out using a kit (Randox^®^, RANDOX Laboratories Ltd., Ireland, UK). Blood plasma concentration of triiodothyronine (T3; nmol/L) was quantified using an enzyme-linked immunosorbent assay (ELISA) test (IMMULITE 1000^®^, Siemens Healthcare Diagnostic Products, Malvern, PA, USA). Blood plasma globulin was determined by subtracting albumin from the total protein concentration. Calculated osmolality (OSM; mmol/L) was determined as defined by Bhagat et al. [30].

### 2.6. Immune Response to Ovalbumin (OVA) Vaccination

The specific antibody response was established by evaluating the response to OVA injection, following the methodology adapted from You et al. [31]. Briefly, the vaccines were prepared by dissolving 15 mg of Quil-A (Purified Saponin Quil-A^®^ Brenntag Biosector, Frederikssund, Denmark) and 15 mg OVA (Chicken egg white albumin, Sigma Catalogue #A5503-5G, Sigma-Aldrich, Spruce Street, NY, USA) in 30 mL of physiological saline. Then 2 mL were delivered intramuscularly, in the shoulder region of the heifers, on days 0 and 14 of the immune response evaluation. To determine the baseline, primary, and secondary responses of OVA specific IgM and IgG1, blood samples were collected prior to vaccination on day 0, prior to the booster on day 14 and at day 21, respectively.

The baseline and specific antibody response was quantified using a modified antigen specific IgM and IgG1 ELISA [32]. The bovine IgG1 response to OVA was evaluated by modified ELISA [17]. Pooled positive control sera from day 21 samplings and individual serum samples, were diluted to 1:1600 and 1:3200 in sample conjugate buffer (PBS containing 1.5% Tween 20 and 0.3 M NaCl). Likewise, alkaline phosphatase-conjugated sheep anti-bovine IgG1 (Cedarlane, Burlinton, ON, Canada) was diluted to 1:4000 in sample conjugate buffer. All control and individual samples were added in triplicate. The plates were read on the Wallac1420 VICTOR 3^®^ Multilabel Counter (PerkinElmer, Waltham, MA, USA) at 405 nm, and the optical density (OD) was obtained.

The OVA-specific bovine IgM was measured in a similar way to IgG1, except the control sera, individual serum samples and the alkaline phosphatase-conjugated rabbit anti-bovine IgM detection antibody (Cedarlane) were diluted to 1:100, 1:200, and 1:2000, respectively, in sample conjugate buffer. Coated buffer wells containing serum were also included for each individual sample to account for non-specific IgM binding. All OD readings were normalized across plates using the correction factor (*CF*) [31] calculated as:

$$CF=\frac{\mathrm{overall}\;\mathrm{mean}\;\mathrm{OD}\;\mathrm{of}\;\mathrm{positive}\;\mathrm{control}\;\mathrm{from}\;\mathrm{all}\;\mathrm{tested}\;\mathrm{plates}\;\left(100x+200x\;\mathrm{for}\;\mathrm{IgM}\;\mathrm{OR}\;1600x+3200x\;\mathrm{for}\;\mathrm{IgG}1\right)}{\mathrm{actual}\;\mathrm{mean}\;\mathrm{OD}\;\mathrm{of}\;\mathrm{positive}\;\mathrm{control}\;\mathrm{from}\;\mathrm{individual}\;\mathrm{plate}\;}$$

To measure the true OD for IgM, the non-specific binding OD was subtracted from individual sample OD readings. Therefore, all OD readings for each sample were corrected before statistical analysis. For OVA-specific antibody ELISA, the respective inter-assay coefficients of variations were 7.6% for IgG1 and 6.9% for IgM. The adjusted primary and secondary response values of IgG1 and IgM were calculated by subtracting the baseline immunoglobulin concentration from the corresponding primary or secondary response concentrations.

### 2.7. Statistical Analyses

Data were analyzed using the SAS software (SAS version 9.4^®^, SAS Institute Inc., Cary, NC, USA). Normality was tested by the univariate procedure and transformations were completed where necessary. The general linear model (GLM) select procedure was used to determine the optimal linear model for the productive performance, CBC, and immune parameters for both the heifer calves and pregnant heifers. This procedure determined that breed composition was an effect to be included in the evaluation of productive performance measures, but not for CBC and immune response parameters in either population of heifers. Heifer calves were split into two groups based on RFI: efficient (*n* = 54; average RFI = −0.83 kg/day), and inefficient (*n* = 53; average RFI = 0.85 kg/day). Similarly, pregnant heifers were split into efficient (*n* = 16; average RFI = −1.02 kg/day), and inefficient (*n* = 15; average RFI = 1.06 kg/day) groups. All parameters were compared across feed efficiency groups in each population of heifers. Preliminary results for immunoglobulin response suggested a potential distinction between extreme groups for feed efficiency when populations were divided into thirds. Therefore, heifer calves were also classified as efficient (*n* = 36; average RFI = −1.15 kg/day), average (*n* = 36; average RFI = 0.01 kg/day), and inefficient (*n* = 35; average RFI = 1.14 kg/day), and the pregnant heifers as efficient (*n* = 11; average RFI = −1.18 kg/day), average (*n* = 10; average RFI = −0.39 kg/day), and inefficient (*n* = 10; average RFI = 1.64 kg/day) for this particular class of blood analytes.

The GLM procedure model used for productive performance traits between feed efficiency groups was the following:

$$Y_{ijk}=\mu +\alpha_{i}+B_{1}\left(\mathrm{AN}\right)+B_{2}\left(\mathrm{SM}\right)+B_{3}\left(\mathrm{LI}\right)+B_{4}\left(\mathrm{HE}\right)+B_{5}\left(\mathrm{ST}\right)+B_{6}\left(\mathrm{OT}\right)+\varepsilon_{ijk}$$

where $Y_{ijk}$ is the dependent variable measured on the *k*-th animal; μ is the overall mean, $\alpha_{i}$ is the fixed effect of feed efficiency group (*j* = 1, 2, 3); *β*_1_, *β*_2_, *β*_3_, *β*_4_, *β*_5_ and *β*_6_ are the coefficients of the fixed multiple linear regression on breed composition for Angus (AN), Simmental (SM), Limousin (LI), Hereford (HE), Shorthorn (ST) and other breeds (OT), and $\varepsilon_{ijk}$ is the random residual error associated with the assessment made on the *k*-th heifer, belonging to the *i*-th feed efficiency group and with the *j*-th breed composition. A model excluding breed composition was used for the CBC and immune response parameters. Similarly, the least square means of CBC and immune response parameters were also determined to compare heifers in relation to developmental stage (heifer calves vs. pregnant heifers).

Least square means between feed efficiency groups were also compared for repeated measures, as part of the metabolic profile dataset. Preliminary analysis also confirmed a lack of significant effect of breed composition; therefore, the fixed effect of breed was not included in the model below:

(1) $$Y_{ijk}=\mu +\alpha_{i}+\beta_{j}+\varepsilon_{ijk}$$

where $Y_{ijk}$ is the dependent variable (blood analytes) measured on the *k*-th animal; μ is the overall mean, $\alpha_{i}$ is the fixed effect of day of sampling (*i* = 1, 2, 3, 4, 5), $\beta_{j}$ is the fixed effect of the feed efficiency group (*j* = 1, 2) and $\varepsilon_{ijk}$ is the random residual error associated with the assessment made on the *i*-th day in the *k*-th heifer belonging to the *j*-th feed efficiency group. Similarly, the least square means of the repeated measures assessed for metabolic profile were also determined to compare heifers in relation to developmental stage (heifer calf vs. pregnant heifer).

The regression procedure with the backward selection option was used to identify the most relevant variables, and determine the amount the variation in RFI explained by each blood analyte that significantly differed between efficient and inefficient heifers within each developmental stage. The least square means of variables determined by repeated measures were calculated to conduct the regression analysis. Both linear and quadratic terms were tested for each of the selected variables, and in the case of a quadratic effect (Q), the linear (L) term was also maintained in the multiple regression model.

The least square means comparison analysis conducted using the general linear model and mixed model were performed via the Scheffé test. Transformed data were back-transformed and confidence intervals of the least square means were calculated at 95% limits. For all analyses, results were considered significant when *p* ≤ 0.05, and a trend towards significance when 0.10 ≥ *p* > 0.05.

## 3. Results

The descriptive statistics and least square means of productive performance traits by feed efficiency groups for heifer calves and pregnant heifers are shown in Table 1. In both populations of heifers, feed efficient animals demonstrated reduced DMI and FG without affecting indicators of body fatness and leanness. Efficient heifer calves and pregnant heifers demonstrated the potential to consume 303 kg and 358 kg less feed (dry matter basis) yearly, respectively, in comparison to inefficient heifers. The retrospective assessment of days in gestation resulted in an average of 137 ± 78.3 days at the start of performance test for the 31 pregnant heifers; efficient and inefficient pregnant heifers did not differ for days in gestation (least square means (confidence interval), 118 (78.9, 157) vs. 157 (117, 198) days (*p* = 0.16) respectively.

The CBC parameters at the start and end of the performance evaluation, by feed efficiency groups for the heifer calves and pregnant heifers, are shown in Table 2. Feed-efficient heifer calves showed a trend toward a lower MCH (*p* = 0.08) at the start, and demonstrated significantly lower MCH at the end of the testing period. Results from efficient heifer calves also showed a trend toward lower MCV (*p* = 0.09) at the end of the performance test. Results from efficient pregnant heifers suggested a trend toward lower MCV (*p* = 0.07) at the start of the test. Similarly, efficient pregnant heifers had suggested lower MCH at both the start (*p* = 0.09) and the end (*p* = 0.06) of the test. The CBC from efficient pregnant heifers at the start of the performance test suggested a higher WBC (*p* = 0.07). Efficient heifer calves and pregnant heifers showed a greater abundance of lymphocytes and fewer segmented neutrophils, compared to the inefficient heifers, at the start and end of the performance evaluation. Total red blood cell count, hematocrit and hemoglobin content did not differ by feed efficiency group for either heifer calves or pregnant heifers at the start or end of the performance evaluation.

**Figure 1:**
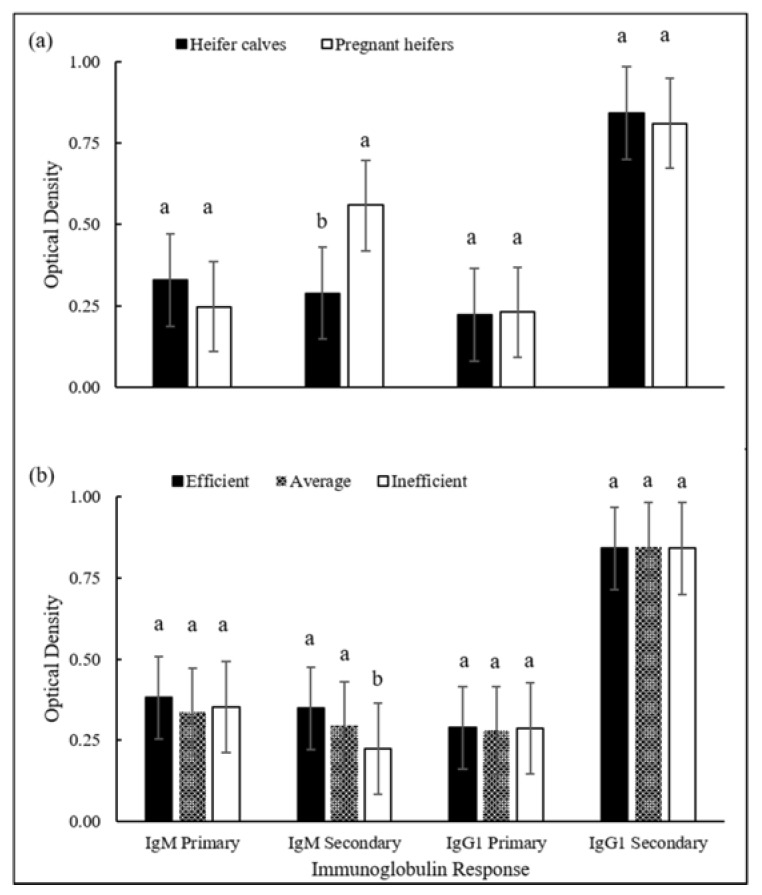
Specific immune response of immunoglobulins M (IgM) and G1 (IgG1) to ovalbumin in beef heifers; (**a**) heifer calves and pregnant heifers; and (**b**) heifer calves by feed efficiency groups (efficient, average and inefficient). Differing superscript denotes *p* < 0.05, according to the Scheffé multiple comparison test and error bars are the 95% confidence interval.

Immunoglobulin responses to OVA of the heifer calves and the pregnant heifers, as well as heifer calves’ immunoglobulin response according to feed efficiency grouping in thirds is shown in Figure 1. When comparing the heifer populations, the secondary response of IgM in the pregnant heifers was greater than that observed in heifer calves, with no differences for IgG1 (Figure 1a). The secondary IgM response was 60.9% lower in the heifer calves and 13.9% lower in the pregnant heifers, compared to the primary IgM response to OVA. Conversely, the secondary IgG1 response was 98.3% higher in the heifer calves and 86.7% higher in the pregnant heifers, by comparison with the primary IgG1 response to OVA. Immunoglobulin response, when compared by thirds, exhibited a greater secondary IgM response in more feed-efficient heifer calves when compared to less feed-efficient heifer calves (Figure 1b).

The blood analysis by developmental stage of the heifers are shown in Table 3. Heifer calves showed greater levels of hematocrit, platelets, RBC, WBC, CK, cholesterol, glucose, potassium and phosphorus, when compared to the pregnant heifers. Pregnant heifers showed greater MCH, MCV, ALP, CO_2_, creatinine, globulin and chloride concentrations than the heifer calves.

The metabolite concentration by feed efficiency group for heifer calves and pregnant heifers are shown in Table 4 and Table 5, respectively. Efficient heifer calves had a greater concentration of phosphorus and potassium, and decreased ALP over the performance evaluation compared to the inefficient heifer calves. Efficient heifer calves also tended to have a lower concentration of T3 during the performance evaluation (*p* = 0.06), compared to the efficient heifer calves. Efficient pregnant heifers showed lower concentrations of cholesterol, lower globulin, increased NEFA, increased ALP, increased creatinine and tended to have higher calcium (*p* = 0.06) compared to the inefficient pregnant heifers.

**Figure 2:**
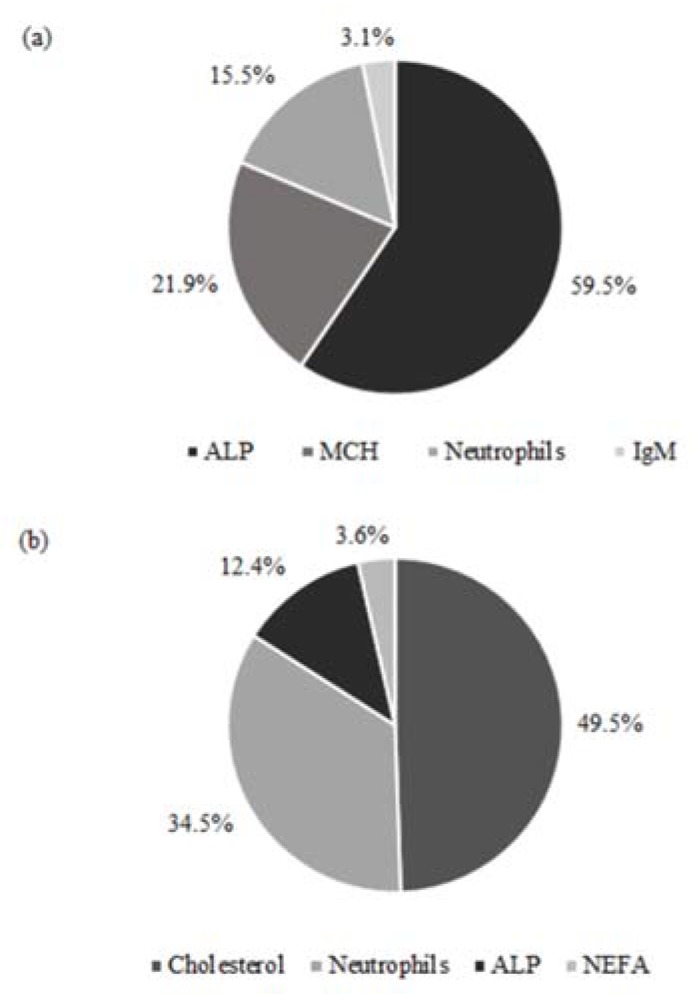
Contribution of the explained variation of residual feed intake by blood analytes. (**a**) heifer calves and (**b**) pregnant heifers. ALP: Alkaline phosphatase; MCH: mean cell hemoglobin. IgM: IgM secondary response; Neutrophils: segmented neutrophils and; NEFA: Non-esterified fatty acids.

The relative contribution to the explained variability of RFI in each of the populations of heifers evaluated is detailed in Figure 2. In the case of heifer calves, the selected parameters (ALP (L), MCH (Q), segmented neutrophils (Q) and IgM secondary (L)) represented a R^2^ = 0.25 and an adjusted R^2^ = 0.17 (*p* = 0.07). In the case of yearling heifers, the selected parameters (ALP (L), cholesterol (L), segmented neutrophils (Q) and NEFA (L)) represented a R^2^ = 0.38 and an adjusted R^2^ = 0.26 (*p* = 0.01). In both populations, ALP and segmented neutrophils accounted for a large proportion of the explained variation.

## 4. Discussion

The feeding regime, the diversity of breed composition and the age span within each developmental stage of the heifers in our study are a typical representation of the grass-fed beef farming systems in Atlantic Canada, which reinforces the practical relevance of our findings. Our study was not designed to produce inferences across different breeds, but these effects were isolated from the productive performance results reported. The difference in feed intake for the same rate of body weight gain, body composition and weight observed when comparing efficient and inefficient heifers, illustrates the possibility of reducing feed costs without hindering productivity. Improvements in the efficiency of feed utilization within the cow-calf herd are important to improve feed efficiency across all production stages given the moderate heritability of RFI [33]. The close association of RFI and the underlying biology of feed efficiency is also a positive aspect to further our understanding and foster the development of biomarkers, as indicated in our results. The CBC, biochemistry and hormonal determinations are used in clinical pathology to infer about health status. In this regard, all our heifers were considered healthy throughout the study. Beyond this use, blood analyses have been related to energy metabolism [6,7,10], developmental stage [7,26] and appear to be associated with feed efficiency in heifers [7,8,9]. Our study (Figure 2) highlights differences in the association between blood analytes and feed efficiency between heifer calves and pregnant heifers, and also identifies similarities across developmental stages that may represent robust traits for the development of feed efficiency proxies, such as levels of ALP and the neutrophils count. Furthermore, this global analysis (Figure 2) highlights the biological differences of factors determining feed efficiency that exist across developmental stages, which are also evidenced throughout the different classes of blood analytes studied. In general, these dissimilarities are opportunities to consider the robustness and suitability of potential proxies for further development towards industry application.

Due to technical issues, the CBC analysis was only carried out at the start and end of the performance test. Even with this limitation, interesting results were observed, including the indication that lymphocytes and segmented neutrophils could work as a proxy for feed efficiency in either developmental stage of the heifers, and the consistence of MCH to infer about feed efficiency even when sampled 124 days apart. The observed trend of reduced MCH and MCV was also found in feed efficient crossbred steers [10]. It has been shown that inefficient animals produce more radiant heat than efficient animals [3], which is associated with increased oxygen consumption [34]. This phenomenon is typically seen in athletic animals, which exhibit greater resting MCH and MCV due to increased oxygen requirements [11]. This suggests that animals with greater oxygen demands have a greater concentration of hemoglobin and higher corpuscular volume per erythrocyte. There were no differences in RBC between feed efficiency groups yet both the efficient heifer calves and pregnant heifers tended to exhibit lower MCH and MCV, which implies that inefficient heifers have greater oxygen requirements to sustain service functions. This increased oxygen demand may be associated with an increased basal metabolic rate and, consequently, reduced feed efficiency [1]. The greater MCH and MCV, and reduced RBC shown by the pregnant heifers in comparison to heifer calves may be due to a greater oxygen requirement related to the metabolic demands of pregnancy [35] and physiological age [1]. This is also supported by the observation of the higher plasma concentration of CO_2_ in pregnant heifers.

We suggest that the greater concentration of lymphocytes in the efficient heifers may be related to the presence of more readily available oxygen because of decreased energy requirements. During their quiescent state, lymphocytes depend on oxidative phosphorylation to generate ATP [36]. Thus, feed-efficient heifers with lower oxygen demands may have more oxygen available for oxidative phosphorylation and a preferable condition for lymphocytes, leading to an increased lymphocyte abundance. This could also contribute to better the humoral defense system suggested by our immune challenge on the feed-efficient heifer calves. Conversely, the higher abundance of segmented neutrophils in inefficient heifers may be related to a higher susceptibility to stress, as supported by some studies [37]. As reviewed by Colditz [37], increased segmented neutrophil abundance in mildly stressed cattle is thought to be a result of cortisol release, causing demargination of neutrophils from blood vessel walls following *β*-adrenergic stimulation. This explanation is supported by another study where beef heifers experiencing stress exhibited a reduced FG, and a greater abundance of neutrophils [38]. We understand that this is a reasonable explanation, based on classical literature on factors affecting feed intake and energy metabolism in ruminants [1]. The differences revealed between leukocyte subpopulations could be related to mechanisms involved with the innate immune system. During a neutrophil inflammatory response, increased oxygen consumption results in greater energy expenditure [37]. Thus, the higher abundance of segmented neutrophils in both inefficient heifer calves and pregnant heifers may be related to increased basal energy requirements. Further research on CBC parameters under a more frequent sampling routine is needed to clarify the evidence pointed out in our study. It seems that research should focus on oxygen transport and in the differentiation and biology of white blood cells.

In relation to the humoral defense, the IgM production in response to a booster tended to be equal to, or lower than during the primary response; this was replicated in our study by the pregnant heifers and heifer calves. Conversely, the secondary response of IgG1 tends to be greater [16] as also shown by both populations of heifers in our study. The distinct developmental stages of the pregnant heifers and heifer calves could be related to the differences in IgM. As cattle reach maturity, there is an increased capacity to produce immunoglobulins, which is shown by the abundance of immunoglobulin in the colostrum when comparing younger and older dairy cows [39]. Moreover, the greater secondary response of IgM in the feed-efficient heifers over the inefficient heifers, with the population segregated into thirds based on RFI (Figure 1), could be the result of a superior adaptive immune system and may be related to improved productive performance, which has been shown in other species including poultry [40] and swine [41]. Similar to our results, Lawrence et al. [9] found no differences between RFI categories and total IgG, which includes the IgG1, concentrations in pregnant heifers.

Alkaline phosphatase catalyzes the liberation of inorganic phosphate from phosphate esters and is present in many body tissues including liver, bone and placenta [42]. Previous studies have shown an indirect correlation with ALP and feed intake [43], feed efficiency [6] and with growth rate in cattle [44]. Since the heifer calves in our study were at a stage of rapid growth and development, the increased concentration of ALP in feed inefficient heifer calves may be related to an increased metabolic demand supporting production. Conversely, the higher concentration of ALP in the efficient pregnant heifers may be related to the uterine production of the placental isoform of ALP [45]. It has been demonstrated that insulin-like growth factors that regulate fetal growth become more active following de-phosphorylation by placental ALP [46]. This suggests that higher feed efficiency may be related to an increased supply of provisional resources to the growing fetus. Moreover, the placental production of ALP increases with length of gestation in cows [27]; thus, the difference shown in ALP concentrations according to the developmental stage of the heifers may be related to shifts in energy metabolism during pregnancy that support fetal growth.

Circulating concentrations of NEFA are elevated during late pregnancy, even when energy requirements are met [47]. The greater concentrations of NEFA demonstrated by the efficient pregnant heifers towards the end of the performance evaluation could be related to differences in fat mobilization required to support oxidative metabolism in the maternal tissues [48]. On the other hand, lower cholesterol concentrations in the efficient pregnant heifers may be related to differences in cholesterol metabolism and diminished lipogenesis. Other studies in young bulls [6] and feedlot steers [49] reported reduced cholesterol levels in cattle with higher feed efficiency, supporting our results. This observation could relate to a down-regulation of enzymes involved in the cholesterol synthesis, or a reduction in substrate availability because acetate is required to generate reducing equivalents, or an increase in biliary excretion of cholesterol [50]. However, this remains to be further investigated.

The higher blood plasma globulin concentration in inefficient pregnant heifers may be associated with a greater stress response in these animals. Stressors such as extreme weather [51], and stocking rate [52] are capable of increasing plasma globulin in cattle, which could have contributed to the higher levels of globulin in the pregnant heifers in our study. This observation suggests that the inefficient pregnant heifers had less ability to cope with stressors, negatively influencing their productivity [53]. This also suggests that environmental conditions could be used as a model to identify and refine proxies for efficiency. For instance, further research varying stocking rate and accounting for weather conditions could be used to explore the feasibility of globulin as a definitive proxy for feed efficiency.

The higher concentration of creatinine in efficient pregnant heifers could be related to their age [51]. Greater concentrations of creatinine in feed-efficient pregnant heifers were also observed by Lawrence et al. [9]. The higher concentrations of creatinine observed in the efficient pregnant heifers could indicate greater protein turnover in the muscle tissue [54]. In the same way, the higher concentration of CK observed in the heifer calves in comparison to pregnant heifers may be related to the age of the heifers. This is supported by the findings of Gonano et al. [7] who studied CK in beef heifers across different ages.

Efficient heifer calves and the overall heifer calf group exhibited greater concentrations of blood plasma metabolic ions; phosphorus and potassium, which may be associated with stage of growth. Potassium in the blood decreases when the rate of protein synthesis within the cell increases. This has been suggested by the inhibitory effects of potassium deficiency on growth and protein synthesis in skeletal muscle [55]. Reduced concentrations of potassium in feed-inefficient heifer calves may suggest increased protein synthesis, possibly due to increased protein deposition, mobilization and turnover [54]. In fact, all energy-requiring processes that could increase basal energy expenditure in inefficient animals may be related to potassium metabolism. While potassium levels are within the normal range for these animals, the significance of increased blood plasma potassium levels in efficient animals may relate to increased nutrient absorption and may influence ion exchange as part of the sodium–potassium cellular pump, which will have a major effect on feed efficiency [50].

The greater concentration of phosphorus in efficient heifer calves may indicate a greater availability of phosphorus for growth and energy metabolism. Blood plasma phosphorus also contributes to the production of the muscle storage molecules including creatine phosphate and ATP [56], providing a more readily available energy source in the post-absorptive state of efficient heifer calves. Beyond one year of age, blood plasma concentrations of phosphorus have been shown to decrease in beef cattle [26], which may explain the higher phosphorus concentrations for the heifer calves relative to the pregnant heifers.

A higher concentration of T3 is related to feed inefficiency in non-lactating cows [22] and young beef bulls [6]. Thyroid hormones regulate anabolism and catabolism that result in fluctuations in heat production [57]. The tendency for higher concentration of T3 in the feed-inefficient heifer calves and greater abundance of MCH suggest an increased metabolic rate, thereby increasing the oxygen demands and reducing feed efficiency. Triiodothyronine may also be related to the greater abundance of ALP in the feed-inefficient heifer calves. When applied to rat osteoblasts in vitro, T3 increased the activity of ALP [58], suggesting T3 may also be related to the greater abundance of ALP in feed-inefficient heifer calves. It is also documented that during growth, T3 has a synergistic relationship with the growth hormone in heifers [59], supporting the argument of metabolic rate differences between heifer calves of distinct feed efficiency classifications. However, it is important to consider that the linkages between feed efficiency and T3 are not fully understood. Our study demonstrated a potential direct relationship between T3 and feed efficiency in pregnant heifers, and Bourgon et al. [6] demonstrated an interaction between age and feed efficiency, supporting a positive association during earlier ages and an antagonism at later ages in young beef bulls.

## 5. Conclusions

Feed-efficient heifer calves appear to have lower oxygen requirements and the same is suggested for efficient pregnant heifers, which is consonant with increased energy conservation. Results support a lack of antagonistic associations between improved feed efficiency and humoral defense as indicated by IgG1 concentrations, and a potential synergistic association as indicated by IgM in heifer calves. Cellular defense is linked with feed efficiency, as indicated by the direct and indirect associations between lymphocytes and segmented neutrophils, respectively, in both populations of heifers. A higher concentration of potassium and phosphorus in the efficient heifer calves may explain the greater efficiency during stages of growth and development. Efficient pregnant heifers have reduced concentrations of cholesterol and globulin, which indicate changes in liver metabolism. The developmental stage should be considered when measuring blood analytes identified as proxies for feed efficiency, based on the major differences observed between the heifer calves and pregnant heifers. The consistence observed for ALP levels and neutrophils count as important factors explaining the variation in feed efficiency, and demonstrates a remarkable robustness for these determinations. Further studies are warranted to evaluate the repeatability of our results in other herds, and to optimize a sampling protocol. It seems that blood analytes should be chosen according to developmental stage and the frequency of sampling urges to be optimized. This study has practical implications for efforts to reduce the need and/or shortening animal performance evaluation trials for the purpose of superior breeding stock selection.

## Figures and Tables

**Table 1:** Descriptive statistics (mean and standard deviation, SD) and least square means (confidence interval) of productivity traits by developmental stage of the heifers and feed efficiency group.

Stage/Variable (Unit)	Mean ± SD	Efficient	Inefficient	*p*-Value
Heifer calves		(*n* = 54)	(*n* = 53)	
Residual feed intake (kg DMI/day)	0.00 ± 1.07	−0.83 (−1.01, −0.66)	0.85 (0.67, 1.03)	<0.01
Average daily gain (kg/day)	0.73 ± 0.16	0.71 (0.67, 0.76)	0.74 (0.70, 0.79)	0.35
Dry matter intake (DMI; kg/day)	6.22 ± 1.29	5.45 (5.17, 5.73)	7.01 (6.73, 7.30)	<0.01
Feed to gain ratio	8.80 ± 2.18	7.92 (7.38, 8.46)	9.70 (9.15, 10.2)	<0.01
Average body weight (kg)	303 ± 40.4	300 (289, 311)	308 (297, 319)	0.33
Average rib eye area (cm^2^)	40.7 ± 5.67	40.2 (38.7, 41.7)	41.1 (39.6, 42.7)	0.58
Average back fat thickness (mm)	1.41 ± 0.85	1.43 (1.20, 1.66)	1.87 (1.30, 1.62)	0.66
Average rump fat thickness (mm)	1.62 ± 1.25	1.52 (1.18, 1.35)	1.72 (1.38, 1.52)	0.71
Average marbling score ^A^ (1–11)	4.00 ± 0.31	4.00 (3.84, 4.12)	4.09 (3.88, 4.14)	0.80
Pregnant heifers		(*n* = 16)	(*n* = 15)	
Residual feed intake (kg DMI/day)	0.00 ± 1.24	−1.02 (−1.45, −0.59)	1.06 (0.61, 1.50)	<0.01
Average daily gain (kg/day)	0.99 ± 0.17	1.01 (0.93, 1.10)	1.02 (0.94, 1.11)	0.86
Dry matter intake (DMI; kg/day)	9.68 ± 1.73	8.64 (7.89, 9.39)	10.93 (10.2, 11.7)	<0.01
Feed to gain ratio	9.78 ± 2.02	8.57 (7.69, 9.45)	10.56 (9.65, 11.47)	<0.01
Average body weight (kg)	480 ± 49.6	480 (455, 505)	496 (470, 522)	0.35
Average rib eye area (cm^2^)	51.1 ± 7.52	50.3 (46.3, 54.3)	53.2 (49.1, 57.3)	0.32
Average back fat thickness (mm)	1.98 ± 1.11	1.82 (1.23, 2.41)	2.27 (1.67, 2.88)	0.30
Average rump fat thickness (mm)	3.20 ± 2.12	3.04 (1.65, 3.67)	3.36 (1.79, 4.03)	0.71
Average marbling score ^A^ (1–11)	4.91 ± 0.58	4.96 (3.68, 4.24)	4.90 (3.61, 4.19)	0.79

^A^ 1: devoid; 11: prime according to Canadian beef quality grade.

**Table 2:** Blood cell count least square means (confidence interval) by feed efficiency group of both developmental stages at the start and end of the test.

Stage/Analyte (Abbreviation; Unit)	Start of Performance Evaluation			End of Performance Evaluation		
	Efficient	Inefficient	*p*-Value	Efficient	Inefficient	*p*-Value
Heifer calves	(*n* = 54)	(*n* = 53)		(*n* = 54)	(*n* = 53)	
Fibrinogen (g/dL)	4.26 (4.00, 4.52)	4.19 (3.93, 4.45)	0.70	3.80 (3.50, 4.09)	3.85 (3.55, 4.15)	0.81
Hematocrit (%)	35.9 (34.9, 37.0)	35.9 (34.8, 37.0)	0.94	35.4 (34.4, 36.4)	36.1 (35.1, 37.1)	0.32
Hemoglobin (mg/dL)	120 (117, 124)	120 (117, 124)	0.90	122 (122, 129)	125 (119, 126)	0.20
Lymphocytes (WBC %)	67.1 (64.4, 69.8)	63.3 (60.6, 66.0)	0.05	62.2 (59.4, 65.1)	60.2 (57.3, 63.1)	0.31
Mean cell hemoglobin (MCH; pg)	14.5 (14.4, 14.7)	14.7 (14.6, 14.9)	0.08	16.0 (15.8, 16.2)	16.4 (16.2, 16.7)	0.01
Mean corpuscular volume (MCV; Hfl)	43.3 (42.8, 43.8)	43.8 (43.3, 44.4)	0.19	46.2 (45.6, 46.9)	47.0 (46.4, 47.7)	0.09
Monocytes (WBC %)	3.61 (3.07, 4.16)	3.53 (3.00, 4.08)	0.83	4.10 (3.44, 4.75)	3.71 (3.04, 4.39)	0.42
Platelets (10^3^ cells/µL)	514 (473, 555)	486 (444, 528)	0.34	417 (385, 449)	430 (398, 463)	0.55
Red blood cells (RBC; 10^6^ cells/µL)	8.29 (8.05, 8.54)	8.19 (7.94, 8.44)	0.56	7.65 (7.43, 7.87)	7.67 (7.44, 7.89)	0.92
Segmented neutrophils (WBC %)	27.1 (24.5, 29.7)	30.9 (28.2, 33.5)	0.05	26.9 (24.5, 29.3)	27.0 (24. 6, 29.4)	0.96
White blood cells (WBC; 10^3^ cells/µL)	10.3 (9.74, 10.9)	10.3 (9.70, 10.9)	0.94	10.1 (9.50, 10.8)	9.81 (9.17, 10.5)	0.48
Pregnant heifers	(*n* = 16)	(*n* = 15)		(*n* = 16)	(*n* = 15)	
Fibrinogen (g/dL)	4.94 (4.35, 5.54)	4.38 (3.69, 5.08)	0.22	3.78 (2.99, 4.57)	4.23 (3.30, 5.16)	0.45
Hematocrit (%)	32.4 (30.7, 34.1)	31.7 (29.9, 33.4)	0.49	35.9 (34.3, 37.4)	36.3 (34.7 37.9)	0.92
Hemoglobin (mg/dL)	116 (110, 122)	113 (108, 120)	0.56	121 (115, 127)	122 (117, 128)	0.72
Lymphocytes (WBC %)	62.3 (57.8, 66.8)	62.1 (57.5, 66.8)	0.96	68.4 (64.5, 72.4)	62.5 (58.5, 66.6)	0.04
Mean cell hemoglobin (MCH; pg)	15.3 (14.8, 15.8)	16.0 (15.4, 16.6)	0.09	16.7 (16.2, 17.2)	17.4 (16.9, 18.0)	0.06
Mean corpuscular volume (MCV; Hfl)	41.9 (40.3, 43.7)	43.9 (42.1, 46.0)	0.07	50.0 (48.4, 51.70)	51.9 (49.9, 53.8)	0.23
Monocytes (WBC %)	3.67 (2.30, 5.03)	3.23 (1.63, 4.84)	0.68	4.65 (3.34, 5.96)	4.46 (2.96, 5.96)	0.85
Platelets (10^3^ cells/µL)	396 (332, 461)	340 (272, 409)	0.23	307 (253, 360)	311 (255, 366)	0.91
Red blood cells (RBC; 10^6^ cells/µL)	7.72 (7.21, 8.23)	7.1 (6.7, 7.7)	0.17	7.23 (6.86, 7.59)	7.03 (6.65, 7.41)	0.45
Segmented neutrophils (WBC %)	30.5 (26.4, 34.6)	31.1 (26.9, 35.4)	0.83	20.0 (16.7, 23.3)	26.6 (23.2, 30.0)	0.01
White blood cells (WBC; 10^3^ cells/µL)	8.94 (8.18, 9.78)	7.96 (7.26, 8.73)	0.07	8.24 (7.33, 9.26)	8.1 (7.12, 9.10)	0.80

**Table 3:** Least square means comparison (confidence interval) of blood cell parameters and plasma metabolites between heifer calves and pregnant heifers.

Traits (Abbreviation; Unit)	Heifer Calves	Pregnant Heifers	*p*-Value
Complete blood cell parameters *			
Fibrinogen (g/dL)	4.03 (3.87, 4.19)	4.34 (4.04, 4.64)	0.07
Hematocrit (%)	35.8 (35.2, 36.4)	34.1 (33.0, 35.1)	0.01
Hemoglobin (mg/dL)	122 (120, 125)	119 (115, 123)	0.15
Lymphocytes (% WBC)	63.3 (61.7, 64.9)	63.9 (60.9, 66.9)	0.72
Mean cell hemoglobin (MCH; pg)	15.4 (15.3, 15.6)	16.2 (15.9, 16.4)	<0.01
Mean corpuscular value (MCV; Hfl)	45.1 (44.7, 45.4)	46.1 (45.4, 46.8)	0.01
Monocytes (% WBC)	3.75 (3.42, 4.09)	4.10 (3.47, 4.72)	0.34
Platelets (10^3^ cells/µL)	462 (440, 484)	336 (296, 377)	<0.01
Red blood cells (RBC; 10^6^ cells/µL)	7.95 (7.81, 8.10)	7.30 (7.03, 7.57)	<0.01
Segmented neutrophils (% WBC)	27.9 (26.5, 29.3)	27.0 (24.4, 29.6)	0.55
White blood cells (WBC; 10^3^ cells/µL)	9.95 (9.60, 10.3)	8.33 (7.79, 8.91)	<0.01
Enzymes			
Alkaline phosphatase (ALP; U/L)	95.1 (88.9, 102)	115 (99.3, 136)	0.02
Aspartate aminotransferase (AST; U/L)	55.0 (53.7, 56.2)	56.9 (54.5, 59.2)	0.17
Creatine kinase (CK; U/L)	171 (164, 178)	153 (142, 166)	0.02
Gamma glutamyl transferase (GGT; U/L)	16.2 (15.8, 16.6)	16.2 (15.4, 17.0)	0.96
Glutamate dehydrogenase (GLDH; U/L)	18.1 (17.0, 19.4)	16.1 (14.2, 18.1)	0.09
Compounds			
Albumin (g/L)	34.0 (33.7, 34.3)	33.8 (33.3, 34.3)	0.56
Albumin: globulin ratio	1.03 (1.01, 1.05)	0.99 (0.96, 1.03)	0.08
Beta hydroxy butarate acid (BHBA; µmol/L)	161 (154, 167)	167 (156, 180)	0.32
Carbon dioxide (CO_2_; mmol/L)	23.4 (23.1, 23.7)	26.2 (25.6, 26.9)	<0.01
Cholesterol (mmol/L)	3.42 (3.31, 3.53)	3.05 (2.85, 3.25)	<0.01
Creatinine (µmol/L)	115 (112, 117)	126 (120, 131)	<0.01
Globulin (g/L)	33.5 (33.0, 34.0)	34.5 (33.7, 35.4)	0.04
Glucose (mmol/L)	4.36 (4.29, 4.43)	3.96 (3.85, 4.08)	<0.01
Non-esterified fatty acids (NEFA: mmol/L)	0.26 (0.25, 0.28)	0.28 (0.25, 0.32)	0.34
Urea (mmol/L)	2.65 (2.58, 2.72)	2.76 (2.63, 2.88)	0.15
Ions			
Anion gap (mmol/L)	27.4 (26.9, 27.8)	27.8 (27.0, 28.7)	0.33
Calcium (mmol/L)	2.40 (2.38, 2.41)	2.39 (2.37, 2.42)	0.84
Chloride (mmol/L)	94.6 (94.3, 94.9)	96.6 (96.0, 97.1)	<0.01
Magnesium (mmol/L)	0.87 (0.86, 0.88)	0.89 (0.87, 0.91)	0.19
Osmolality (mmol/L)	277 (277, 278)	277 (276, 278)	0.39
Potassium (mmol/L)	4.30 (4.26, 4.34)	4.21 (4.13, 4.28)	0.03
Phosphorus (mmol/L)	2.19 (2.15, 2.23)	2.00 (1.93, 2.07)	<0.01
Sodium (mmol/L)	141 (141, 141)	141 (141, 141)	0.94
Hormone			
Triiodothyronine (T3; nmol/L)	1.86 (1.80, 1.91)	1.77 (1.67, 1.87)	0.15

* This class of blood analytes represent least square means of the average combining the start and end of the performance evaluation results. All other classes represent least square means of the repeated measures obtained through five assessments over the 124 days performance test.

**Table 4:** Blood plasma analytes least square means (confidence interval) across the performance evaluation test in heifer calves by feed efficiency group repeatedly sampled.

Traits (Abbreviation; Unit)	Efficient	Inefficient	*p*-Value
Enzymes			
Alkaline phosphatase (ALP; U/L)	86.4 (93.9, 121)	98.6 (103, 131)	0.03
Aspartate aminotransferase (AST; U/L)	56.1 (54.3, 57.9)	55.2 (53.3, 57.0)	0.24
Creatine kinase (CK; U/L)	160 (170, 196)	155 (165, 192)	0.22
Gamma glutamyl transferase (GGT; U/L)	16.5 (15.9, 17.1)	16.4 (15.7, 17.0)	0.76
Glutamate dehydrogenase (GLDH; U/L)	20.4 (17.9, 23.0)	21.6 (19.0, 24.2)	0.55
Compounds			
Albumin (g/L)	34.0 (33.3, 34.2)	33.8 (33.4, 34.2)	0.87
Albumin globulin ratio	1.01 (0.99, 1.04)	1.02 (1.00, 1.05)	0.18
Beta hydroxy butarate acid (BHBA; µmol/L)	155 (153, 169)	156 (153, 168)	0.99
Carbon dioxide (CO_2_; mmol/L)	23.3 (22.9, 23.8)	23.5 (23.0, 23.9)	0.73
Cholesterol (mmol/L)	3.3 (3.3, 3.5)	3.36 (3.31, 3.57)	0.72
Creatinine (µmol/L)	115 (112, 119)	113.2 (109, 116)	0.32
Globulin (g/L)	34.1 (33.3, 34.8)	33.7 (32.9, 34.5)	0.50
Glucose (mmol/L)	4.43 (4.30, 4.57)	4.47 (4.33, 4.61)	0.32
Non-esterified fatty acids (NEFA: mmol/L)	0.35 (0.31, 0.38)	0.31 (0.28, 0.35)	0.15
Urea (mmol/L)	2.64 (2.57, 2.71)	2.65 (2.58, 2.73)	0.79
Ions			
Anion Gap (mmol/L)	27.7 (27.1, 28.3)	27.2 (26.6, 27.8)	0.26
Calcium (mmol/L)	2.39 (2.38, 2.41)	2.40 (2.38, 2.42)	0.55
Chloride (mmol/L)	94.5 (94.2, 94.8)	94.7 (94.4, 95.0)	0.33
Magnesium (mmol/L)	0.87 (0.86, 0.88)	0.88 (0.86, 0.89)	0.44
Osmolality (mmol/L)	277 (277, 278)	277 (277, 278)	0.87
Potassium (mmol/L)	2.23 (2.18, 2.27)	2.16 (2.11, 2.20)	0.03
Phosphorus (mmol/L)	4.36 (4.31, 4.41)	4.24 (4.19, 4.29)	<0.01
Sodium (mmol/L)	141 (141, 141)	141 (141, 141)	0.98
Hormone			
Triiodothyronine (T3; nmol/L)	1.81 (1.75, 1.88)	1.90 (1.83, 1.96)	0.06

**Table 5:** Blood plasma analytes least square means (confidence interval) across the performance evaluation test in pregnant heifers by feed efficiency group repeatedly sampled.

Traits (Abbreviation; Unit)	Efficient	Inefficient	*p*-Value
Enzymes			
Alkaline phosphatase (ALP; U/L)	112 (97.9, 155)	71.2 (62.6, 82.6)	<0.01
Aspartate aminotransferase (AST; U/L)	55.4 (52.6, 58.2)	58.4 (55.5, 61.3)	0.14
Creatine kinase (CK; U/L)	131 (122, 143)	143 (132, 158)	0.14
Gamma glutamyl transferase (GGT; U/L)	16.2 (15.1, 17.4)	16.2 (15.0, 17.4)	0.98
Glutamate dehydrogenase (GLDH; U/L)	14.3 (12.1, 17.0)	16.3 (13.7, 19.3)	0.30
Compounds			
Albumin (g/L)	33.7 (33.2, 34.3)	33.9 (33.3, 34.5)	0.61
Albumin globulin ratio	1.02 (0.98, 1.06)	0.97 (0.93, 1.01)	0.07
Beta hydroxy butarate acid (BHBA; µmol/L)	161 (148, 176)	166 (151, 181)	0.67
Carbon dioxide (CO_2_; mmol/L)	22.9 (22.2, 23.6)	22.3 (21.6, 23.0)	0.22
Cholesterol (mmol/L)	2.68 (2.40, 2.97)	3.42 (3.13, 3.72)	<0.01
Creatinine (µmol/L)	131 (123, 140)	120 (111, 128)	0.05
Globulin (g/L)	33.7 (32.5, 34.7)	35.6 (34.4, 36.7)	0.02
Glucose (mmol/L)	3.96 (3.87, 4.05)	3.93 (3.84, 4.02)	0.62
Non-esterified fatty acids (NEFA: mmol/L)	0.34 (0.30, 0.38)	0.24 (0.21, 0.28)	<0.01
Urea (mmol/L)	2.74 (2.59, 2.89)	2.57 (2.43, 2.72)	0.10
Ions			
Anion Gap (mmol/L)	26.5 (25.8, 27.3)	26.6 (25.8, 27.3)	0.99
Calcium (mmol/L)	2.37 (2.34, 2.40)	2.42 (2.38, 2.45)	0.06
Chloride (mmol/L)	96.6 (95.8, 97.3)	96.5 (95.7, 97.3)	0.95
Magnesium (mmol/L)	0.88 (0.85, 0.91)	0.89 (0.87, 0.92)	0.54
Osmolality (mmol/L)	278 (277, 279)	277 (275, 278)	0.20
Potassium (mmol/L)	4.15 (4.06, 4.24)	4.24 (4.15, 4.34)	0.15
Phosphorus (mmol/L)	2.02 (1.91, 2.14)	1.97 (1.86, 2.09)	0.52
Sodium (mmol/L)	141 (141, 141)	141 (140, 142)	0.20
Hormone			
Triiodothyronine (T3; nmol/L)	1.83 (1.70, 1.95)	1.71 (1.58, 1.84)	0.20
